# Supplementary material for: Are carbon emissions trading and green financial instruments synergistic? -Comprehensive quantitative research based on content analysis
Source: PLoS One. 2024 Mar 7;19(3):e0298601. doi: 10.1371/journal.pone.0298601 (PMC10919601; doi:10.1371/journal.pone.0298601)
Supplement: S1 Data — (ZIP) [file pone.0298601.s001.zip › Data/地级市Z_F工作报告.zip!/▒▒╛⌐/└┤╫╘╣1⁄2╓┌║┼í╛┬φ┐╦╩2╛▌═°í┐_2016.docx]

——2016年1月22日在北京市第十四届人民代表大会第四次会议上

北京市市长 王安顺

各位代表：

　　现在，我代表北京市人民政府，向大会报告政府工作，请予审议，并请市政协各位委员提出意见。

　　一、“十二五”时期经济社会发展回顾

　　“十二五”时期是北京发展具有重大历史意义的五年。在北京改革发展的关键时刻，习近平总书记亲临视察并发表重要讲话，明确了全国政治中心、文化中心、国际交往中心、科技创新中心的城市战略定位，提出了建设国际一流的和谐宜居之都的目标，对做好北京发展和管理工作、推动京津冀协同发展作出了重要指示，指明了新时期首都工作方向。中央把京津冀协同发展确立为重大国家战略，制定了《京津冀协同发展规划纲要》，为首都未来发展描绘了宏伟蓝图和光明前景。首都科学发展开启了新征程。

　　五年来，在党中央、国务院坚强领导下，在中共北京市委直接领导下，在市人大及其常委会监督支持下，我们坚决贯彻落实中央决策部署，坚持稳中求进工作总基调，牢牢把握首都城市战略定位，积极推动京津冀协同发展，扎实做好稳增长、促改革、调结构、惠民生各项工作，保持经济社会持续健康发展，较好地完成了“十二五”规划目标任务，北京综合经济实力、科技创新能力、国际影响力显著增强，向着国际一流的和谐宜居之都迈出了坚实步伐。

　　(一)积极适应经济发展新常态，有效应对经济下行压力，首都经济实力显著增强。更加注重经济发展的质量和效益，在平稳增长中实现了结构更优、质量更高、效益更好的发展。五年来，全市地区生产总值年均增长7.5%，一般公共预算收入年均增长14.9%。人均地区生产总值由2010年的1.1万美元增加到1.7万美元；社会消费品零售额由6300多亿元增加到1万亿元以上，服务性消费占市场消费总额的比重达到44.6%；全社会固定资产投资由5200多亿元增加到近8000亿元。城镇居民人均收入年均实际增长7.2%；农村居民人均收入年均实际增长7.8%，增速持续高于城镇居民。居民消费价格涨幅控制在预期目标以内。万元地区生产总值能耗、水耗和二氧化碳排放分别累计下降25%、24%和27%左右。顺利完成北京市第三次全国经济普查。

　　京津冀协同发展实现良好开局。坚决贯彻落实《京津冀协同发展规划纲要》，制定实施我市贯彻意见。有序疏解非首都功能，发布修订新增产业的禁止和限制目录，不予办理的工商登记业务累计1.3万件；关停退出一般制造和污染企业1006家，退出低端市场228家。大力实施交通、生态、产业三个重点领域率先突破工作方案，京津冀城际铁路网规划正式上报，京昆高速北京段建成通车，北京新机场主体工程开工建设，一批重大区域生态工程顺利推进，曹妃甸协同发展示范区等合作园区建设步伐加快。京津冀通关一体化成效显著，教育、医疗、旅游等领域合作有序开展。

　　(二)实施创新驱动，着力转型发展，经济结构高端化更趋明显。市级一般公共预算科技文化支出比“十一五”期间增长1.1倍，北京地区综合科技进步水平居全国首位，创新驱动发展格局初步形成。积极推动中关村国家自主创新示范区建设，落实“1+6”、“新四条”等一系列先行先试政策，出台加快全国科技创新中心建设的意见，发布实施“京校十条”、“京科九条”，开展科技成果使用、处置、收益管理改革和股权激励试点。不断优化创新创业生态系统，加快建设人才管理改革试验区和科技金融创新中心，支持众创空间等孵化机构集聚发展，大众创业、万众创新日趋活跃。五年来，自主创新能力不断增强，涌现出一批国际领先的重大科技成果，催生了一批新技术、新产品、新模式、新业态。“十二五”期间，全市专利授权量、技术合同成交额、中关村示范区总收入都实现了翻番。

　　文化创新增添发展新活力。大力培育和践行社会主义核心价值观，持续推进中国特色社会主义和中国梦宣传教育，深入开展精神文明创建活动，积极树立“北京榜样”。出台加强全国文化中心建设实施意见，推动国有文化资产管理体制改革，组建歌华传媒和北京新媒体集团，健全文化要素市场，完善投融资服务体系。文艺创作繁荣发展，18部作品获得全国“五个一工程”奖。制定出台加强基层公共文化建设“1+3”政策文件，实施文化惠民工程，公共文化设施体系进一步健全。加强历史文化名城保护，完成明城墙遗迹等修缮工程。制定实施文化创意产业功能区建设发展规划，文化创意与相关产业融合发展势头良好。

　　高端产业构筑发展新优势。开展石景山国家服务业综合改革试点和中关村现代服务业试点，出台加快首都科技服务业发展意见，实施促进健康、养老、体育产业发展的政策措施，金融、信息、科技服务三大优势产业对经济增长的贡献率超过70%，第三产业比重由75.5%提高到79.8%。制定实施加快发展战略性新兴产业的意见，出台《中国制造2025》北京行动纲要，产业结构加快向高端化迈进。积极推动园区特色化、差异化发展，六大高端产业功能区集聚发展水平进一步提升。

　　(三)统筹城乡发展，优化空间布局，城乡一体化格局基本形成。市级一般公共预算农林水支出比“十一五”期间增长1.2倍，市政府固定资产投资投向郊区的比例超过50%，城乡发展一体化体制机制初步建立。深入开展城市总体规划编制工作，积极谋划未来首都可持续发展蓝图。全力推动市行政副中心建设，研究提出了规划方案，完成一期6平方公里拆迁任务，行政办公区起步区开工建设。积极推进郊区新城建设，重点功能和产业项目加快布局，轨道交通向5个新城延伸。推动42个重点小城镇建设，建成一批新型农村社区，农民享受到了更好的公共服务。城乡结合部50个重点村建设任务基本完成，拆迁还绿14.2平方公里，“一绿”地区6个乡城市化建设试点顺利推进。连续实施两个阶段城南行动计划，城南、西部地区基础设施和生态环境明显改善。

　　农村改革发展扎实推进。深入实施“新三起来”工程，全面完成集体林权制度改革主体任务和集体土地所有权确权颁证，确权承包土地流转比例达到61.7%，集体经营性建设用地入市等改革试点顺利开展。制定实施农业“调转节”意见，高耗水农作物逐步退出，都市型现代农业加快发展。建设美丽乡村1000个，50多万户农宅实施抗震节能改造，村级公益事业专项补助标准提高了50%。实施农村经济薄弱地区发展规划、共同致富行动计划，加大低收入村帮扶力度，低收入农户人均收入年均实际增长10.2%。生态涵养区建成一批特色品牌沟域，搬迁山区农民4.4万人。顺利完成2019年世界园艺博览会园区综合规划、综合交通规划编制和拆迁任务，启动配套基础设施建设。

　　(四)加快破解难题，实施精细管理，“大城市病”治理全面展开。严格落实大气污染防治条例，制定实施清洁空气行动计划，建立区域大气污染联防联控机制，压减燃煤近1400万吨，淘汰全部黄标车，淘汰老旧机动车183.2万辆，细颗粒物浓度比2012年下降15.8%，四项主要污染物减排超额完成目标任务。实施“建管限”综合治堵措施，加大轨道交通建设力度，新增运营里程218公里、总里程达到554公里，城市路网进一步完善。制定实施垃圾、污水处理设施建设两个三年行动计划，新增生活垃圾日处理能力1.2万吨、污水日处理能力120万吨，生活垃圾资源化率提高到55%，再生水年利用量达到9.5亿立方米。完成平原地区造林105万亩，新增城市绿地4850公顷，森林覆盖率、林木绿化率达到41.6%和59%，比2010年分别提高了4.6和6个百分点。

　　制定实施更加严格的人口规模调控工作方案，常住人口比“十一五”期间年均少增43.1万人，年均增速由5%回落到2%。组织开展环境建设精细管理行动，拆除违法建设4611万平方米，架空线入地改造605公里。加强资源能源保障，南水北调中线一期工程建成通水，陕京三线等重要基础设施建成投用，气象监测预测、预报预警水平稳步提升，城市运行保障和防灾减灾能力显著增强。开展石景山区城市管理体制改革试点，夯实基层管理力量，强化部门联动综合执法，网格化社会服务管理体系覆盖90%以上的街道和社区。

　　(五)创新社会治理，增进民生福祉，和谐稳定良好局面更加巩固。增投入、建机制、兜底线，市级一般公共预算民生支出比“十一五”期间增长1.2倍，累计办理153项重要民生实事，解决了一批关系群众切身利益的现实问题。实施就业优先战略和更加积极的就业政策，城乡统一的就业体系更加健全，城镇新增就业216.7万人，城镇登记失业率控制在1.6%以内。覆盖城乡全体居民的养老和医疗保障体系进一步完善，“人人享有社会保障”的目标基本实现，六项待遇标准与2010年相比平均增长68%。城乡低保标准实现并轨，适度普惠型社会福利体系基本形成。全面落实居家养老服务条例，统筹推进居家、社区和机构养老服务发展，全市养老床位达12万张。完善残疾人社会保障和服务体系，残疾人事业加快发展。大力实施保障性安居工程，建立“以租为主”的基本住房保障体系，累计建设筹集各类保障房100万套，完成8.3万户棚户区改造和6562万平方米老旧小区综合整治，群众居住条件进一步改善。

　　基本公共服务均等化水平明显提升。全面深化教育综合改革，大力推进素质教育，实施学前教育、中小学建设、市属高校建设三个“三年行动计划”，新建改扩建幼儿园843所、中小学校200所，分别增加学位10万个和19万个。坚持义务教育免试就近入学，推行学区制、九年一贯制、教育集团等模式，优质教育资源覆盖面进一步扩大。深化医药卫生体制改革，积极开展公立医院管办分开、医药分开综合改革试点，建立城乡居民大病保险制度，医保付费制度改革持续推进，公费医疗制度改革基本完成。开展社区卫生服务机构标准化建设，推进社区卫生服务模式改革，建成10个区域医疗中心、43个区域医联体，城乡全覆盖的医疗卫生服务体系不断完善。“单独两孩”政策顺利实施，人口计生服务进一步加强。群众体育事业蓬勃发展，竞技体育水平稳步提升。

　　社会保持和谐稳定。出台深化街道社区管理体制改革意见，“一刻钟社区服务圈”覆盖80%的城市社区。推进四类社会组织直接登记，三级枢纽型社会组织工作体系基本形成，社会工作者和志愿者队伍不断壮大。妇女、儿童事业取得新进展，民族、宗教工作进一步加强。坚持走中国特色军民融合发展道路，军政军民团结更加巩固。实施安全发展战略，严格排查治理安全隐患，亿元地区生产总值生产安全事故死亡率比2010年下降47%。推进食品药品安全监管体制改革，重点食品抽检、药品抽验合格率分别达到98.4%和99.7%。深入开展矛盾纠纷排查化解，创新立体化社会治安防控体系，建立健全反恐防恐工作机制，维护了首都安全稳定。

　　(六)全面深化改革，扩大对外开放，发展活力进一步释放。落实全面深化改革的决定，推出了一系列放权、松绑、减负、惠民的改革措施。深入推进政府职能转变，下大力气简政放权，完成新一轮市政府机构改革，取消和下放439项行政审批事项。优化投资项目审批流程，审批时限由300多个工作日缩短到109个工作日。建成市政务服务中心，40个部门、740项审批事项全面进驻。商事制度改革成效明显，“先照后证”、“三证合一、一照一码”全面推行。出台鼓励社会投资的实施意见，130个市场化试点项目落实社会投资来源。制定实施全面深化市属国资国企改革的意见，开展分类监管等一批改革试点，国有企业重组改制加快推进，国有经济布局不断优化，市属企业利润比2010年增长94.7%。财政预算管理制度改革稳步推进，“营改增”取得积极成效。“新三板”落户北京，挂牌公司超过5000家。削减了56%的政府定价项目，顺利实施水电气热、公交票制票价等价格改革，提高排污收费标准，建立了水环境区域补偿制度，要素配置效率进一步提高。

　　开放型经济发展呈现新局面。成功举办三届京交会，服务贸易总额由798亿美元增加到1200多亿美元。投资促进创新高效，实际利用外资达到130亿美元、境外投资达到95.6亿美元，分别是2010年的2倍和12.5倍。实施72小时过境免签、购物离境退税等政策，口岸经济加快发展。环球主题公园项目引进落地。旅游业总收入比“十一五”期间增长73.7%。开展全国首个服务业扩大开放综合试点，首批33项政策措施全面推进。对外交流交往务实活跃，亚洲基础设施投资银行、丝路基金总部落户北京，成立世界旅游城市联合会，友好城市达到52个。港澳台侨工作成果丰硕。认真做好对口支援和帮扶协作，累计投入资金136.4亿元，完成1771个援助项目。

　　(七)履行首都职责，做好“四个服务”，重大活动服务保障任务圆满完成。按照中央要求和统一部署，高标准做好亚太经合组织第22次领导人非正式会议服务保障工作；圆满完成中国人民抗日战争暨世界反法西斯战争胜利70周年纪念活动服务保障任务；与国家体育总局、河北省密切合作，成功获得2022年冬奥会举办权，北京成为世界上首个既举办过夏奥会，又将举办冬奥会的城市。高水平完成2015年北京田径世锦赛筹办工作，为世界奉献了“一场精彩绝伦的世锦赛”。一系列重大活动服务保障任务圆满完成，得益于中央的坚强领导，得益于各兄弟省区市、驻京部队和社会各界的大力支持，得益于广大市民的热情参与和无私奉献，为我们留下了宝贵的物质财富和精神财富。

　　五年来，我们坚持依法行政、从严治政、廉洁勤政，政府自身建设取得了重要进展。认真执行市人大及其常委会的决议和决定，自觉接受人大工作监督、法律监督和人民政协民主监督，共办理市人大代表议案17件、建议6214件，办理市政协提案5467件。加强政府立法工作，提请市人大常委会审议地方性法规草案38项，制定、修改、废止政府规章68项，行政执法行为进一步规范。大力推进大气污染治理、财政预算决算等17个重点领域信息公开，让群众更加有效地监督政府行为。依法全面履行政府职能，完善绩效管理体系，加强督查和行政问责，政府执行力和公信力进一步提升。严守党的政治纪律和政治规矩，认真开展党的群众路线教育实践活动和“三严三实”专题教育，坚决落实中央八项规定精神和国务院“约法三章”，全面落实各项整改任务，扎实整治“四风”问题。坚定不移推进党风廉政建设和反腐败斗争，加大行政监察和审计监督力度，深入开展“小金库”等专项治理，坚决惩治“为官不为”、“小官贪腐”等问题，严肃查处各类违纪违法案件。

　　回顾过去五年，我们深切感受到，这是北京发展方式加快转变、经济结构优化升级的五年；是改革开放深入推进、市场活力不断释放的五年；是基础设施更加完善、城乡统筹一体发展的五年；是生态建设大力推进、城市治理扎实攻坚的五年；是社会事业全面进步、人民得到更多实惠的五年。五年的成就来之不易，这是党中央、国务院坚强领导，中共北京市委直接领导，全市人民团结奋斗的结果，是全国各族人民大力帮助，社会各界关心支持的结果。在这里，我代表北京市人民政府，向全市各族人民，向人大代表、政协委员，向各民主党派、各人民团体和各界人士，向中央各部门、各兄弟省区市，向驻京解放军和武警部队官兵，向所有关心支持首都建设的港澳台同胞、海外侨胞和国际友人，表示衷心的感谢！

　　各位代表，五年的团结奋斗，使我们进一步深化了对做好首都工作的认识，主要体会有：

　　第一，必须始终坚持首都城市战略定位。把落实“四个中心”战略定位作为衡量发展的根本标尺，牢固树立首善标准，认真履行首都职责，提高“四个服务”水平，科学谋划和推动首都建设发展，使城市发展与战略定位相适应、相一致、相协调，在服务国家大局中提升首都发展水平。

　　第二，必须始终坚持改革创新。以改革创新为根本动力，坚持问题导向，不失时机地推出各项改革举措，切实破除制约发展的体制机制障碍；把创新摆在发展全局的核心位置，深入实施创新驱动发展战略，着力激发创新创业活力，发挥创新激励经济增长的乘数效应，率先形成以创新为主要引领和支撑的经济体系和发展模式。

　　第三，必须始终坚持城乡区域协调发展。把协同发展、协调发展作为治理“大城市病”、实现城市可持续发展的必由之路，有序疏解非首都功能，优化提升首都核心功能，加快形成区域协同发展新格局；着眼于城乡经济社会一体化发展，优化城市发展布局和要素配置，逐步缩小城乡发展差距，促进均衡协调发展。

　　第四，必须始终坚持走生态文明之路。坚持节约资源和保护环境的基本国策，坚守生态保护红线、环境质量底线、资源消耗上限，加大污染治理和生态建设力度，着力补齐短板，不断提升城市治理能力，缓解人口资源环境矛盾，提高绿色发展水平。

　　第五，必须始终坚持人民主体地位。以人民为中心，坚定不移走群众路线，把增进市民福祉、促进人的全面发展作为工作的出发点和落脚点，充分调动人民的积极性、主动性和创造性，让全体市民更好地发挥主体作用，共同把首都建设好、发展好、管理好。

　　我们也清醒认识到，发展中不平衡、不协调、不可持续的问题依然存在，前进道路上还有不少困难和挑战。主要是：人口资源环境矛盾突出，治理大气污染、交通拥堵等“大城市病”还需要下更大力气；科技、文化资源优势发挥不够，新旧动力转换接续需要加快推进，转方式、调结构、稳增长还需要下更大功夫；城市服务管理水平和文明程度还不够高，法治化、精细化管理亟待加强；教育、医疗、养老等公共服务供给不足和配置不均衡问题仍然存在，民生保障需要继续提高水平；政府工作还存在不足，一些领域的事中事后监管不到位，少数政府工作人员不作为、乱作为现象依然存在，面对新形势不适应、不会为、不善为的问题比较突出，政府系统勤政廉政建设需要常抓不懈。我们将以高度的责任感和使命感，迎难而上、毫不懈怠，加倍努力工作，扎实解决问题，绝不辜负人民的期望和重托。

　　二、“十三五”时期的主要目标和任务

　　“十三五”时期是我国全面建成小康社会的决胜阶段，也是落实首都城市战略定位、加快建设国际一流的和谐宜居之都的关键阶段。市委十一届八次全会确定了“十三五”时期北京发展的指导思想：高举中国特色社会主义伟大旗帜，全面贯彻党的十八大和十八届三中、四中、五中全会精神，以马克思列宁主义、毛泽东思想、邓小平理论、“三个代表”重要思想、科学发展观为指导，深入贯彻习近平总书记系列重要讲话精神，坚持“四个全面”战略布局，坚持发展是第一要务，牢固树立创新、协调、绿色、开放、共享的发展理念，牢牢把握首都城市战略定位，深入实施京津冀协同发展战略，以有序疏解非首都功能、治理“大城市病”为重点任务，以提升发展的质量和效益为中心，加快形成引领经济发展新常态、实现城市可持续发展的体制机制和发展方式，统筹推进经济建设、政治建设、文化建设、社会建设、生态文明建设和党的建设，率先全面建成小康社会，在建设国际一流的和谐宜居之都上取得重大进展，奋力谱写中华民族伟大复兴中国梦的北京篇章。

　　遵循这一指导思想，紧密衔接《京津冀协同发展规划纲要》确定的目标任务，今后五年全市经济社会发展的主要目标是：

　　——疏解非首都功能取得明显成效。四环路以内区域性的物流基地和专业市场调整退出，部分教育医疗等公共服务机构、行政企事业单位有序疏解迁出。全市常住人口总量控制在2300万人以内，城六区常住人口比2014年下降15%左右，“大城市病”等突出问题得到有效缓解，首都核心功能显著增强。

　　——经济保持中高速增长。在发展质量和效益不断提高的基础上，地区生产总值年均增长6.5%，2020年地区生产总值和城乡居民人均收入比2010年翻一番。主要经济指标平衡协调，劳动生产率和地均产出率大幅提高。三次产业内部结构进一步优化，服务业增加值占地区生产总值比重高于80%，全社会研究与试验发展经费支出占地区生产总值的比重保持6%左右，形成“高精尖”经济结构，成为具有全球影响力的科技创新中心。

　　——人民生活水平和质量普遍提高。公共服务体系更加完善，基本公共服务均等化程度进一步提高。城镇登记失业率低于4%，收入差距缩小，中等收入人口比重上升，“住有所居”水平进一步提高。教育实现现代化，群众健康水平普遍提升，人均期望寿命高于82.4岁。养老助残服务体系更加完善。困难群众基本生活得到有效保障。社会更加安定有序。

　　——市民素质和城市文明程度显著提高。中国梦和社会主义核心价值观更加深入人心，爱国主义、集体主义、社会主义思想广泛弘扬。市民思想道德素质、科学文化素质、健康素质明显提高，热情开朗、大气开放、积极向上、乐于助人的社会风尚更加深厚。文化事业和文化产业蓬勃发展，率先建成公共文化服务体系，全国文化中心地位进一步彰显。

　　——生态环境质量显著提升。生产方式和生活方式绿色、低碳水平进一步提升。单位地区生产总值能耗、水耗持续下降，城乡建设用地控制在2800平方公里以内，碳排放总量得到有效控制。主要污染物排放总量持续削减，生活垃圾无害化处理率达到99.8%以上，污水处理率高于95%，重要河湖水生态系统逐步恢复，森林覆盖率达到44%，环境容量生态空间进一步扩大。

　　——各方面体制机制更加完善。城市治理各领域基础性制度体系基本形成。人民民主更加健全，法治政府基本建成，成为法治中国的首善之区。城乡发展一体化体制机制进一步健全，区域协同发展、统筹利用各级各类资源的体制机制基本建立。开放型经济新体制基本形成。

　　围绕上述指导思想和目标，我市《国民经济和社会发展第十三个五年规划纲要》部署了八个方面的重大任务：一是优化提升首都核心功能；二是建设绿色低碳生态家园；三是提高城市治理水平；四是加快建设全国科技创新中心；五是着力建设全国文化中心；六是持续增进民生福祉；七是全面深化改革开放和加强法治建设；八是全面做好冬奥会筹备工作。

　　三、2016年重点任务

　　2016年是“十三五”开局之年，也是推进结构性改革攻坚之年，做好经济社会发展工作，意义十分重大。过去一年，全市人民齐心协力，锐意进取，迎难而上，经济社会发展取得了新成绩。全市地区生产总值增长6.9%，一般公共预算收入同口径增长12.3%，城乡居民人均收入分别实际增长7%和7.1%，细颗粒物浓度下降6.2%，较好地完成了市十四届人大三次会议确定的主要目标任务，为做好2016年的工作打下了良好基础。

　　2016年政府工作的总体要求是：全面贯彻落实党的十八大和十八届三中、四中、五中全会精神，深入学习贯彻习近平总书记系列重要讲话和对北京工作的重要指示精神，认真贯彻落实中央经济工作会议、中央城市工作会议精神，按照“五位一体”总体布局和“四个全面”战略布局，牢固树立和贯彻落实创新、协调、绿色、开放、共享的发展理念，适应经济发展新常态，坚持改革开放，坚持稳中求进工作总基调，坚持稳增长、调结构、惠民生、防风险，加快疏功能、转方式、治环境、补短板、促协同，着力加强结构性改革，构建“高精尖”经济结构，保持经济运行在合理区间，维护首都社会和谐稳定，努力实现“十三五”良好开局。

　　做好2016年经济社会发展工作，要重点把握好以下几个方面：

　　第一，必须牢固树立创新、协调、绿色、开放、共享的发展理念。把五大发展理念贯穿于经济社会发展全过程和各领域，以发展理念转变引领发展方式转变，以发展方式转变推动发展质量和效益提升，破解发展难题，厚植发展优势，增强发展动力，努力走出一条更高质量、更有效率、更加公平、更可持续的发展新路。

　　第二，必须积极适应、把握、引领新常态。坚持把发展的基点放在创新上，充分发挥首都科技智力资源优势，加快构建“高精尖”经济结构，大力培育新的发展动能，在保持经济中高速增长的同时，切实提高发展的质量和效益。

　　第三，必须着力推进结构性改革。充分发挥市场在资源配置中的决定性作用，更好发挥政府作用，在适度扩大总需求的同时，切实加强供给侧结构性改革，提高供给体系质量和效率，加快形成消费和供给良性互动、需求升级和产业升级共进的格局。

　　第四，必须大力提高城市工作水平。贯彻落实“五个统筹”的要求，准确把握超大型城市发展规律，转变城市发展方式，完善城市治理体系，提高城市治理能力，集中力量解决“大城市病”等突出问题，不断提升城市环境质量、人民生活质量和城市竞争力。

　　综合各方面因素，全市经济社会发展的主要预期目标是：地区生产总值增长6.5%；一般公共预算收入增长6.5%以上；居民消费价格涨幅控制在3.5%左右；城镇登记失业率控制在3%以内；城乡居民可支配收入增长与经济增长同步；万元地区生产总值能耗、水耗和二氧化碳排放分别下降3.5%、3%左右和4%，细颗粒物浓度下降5%左右。

　　为实现上述目标，重点抓好以下几方面工作：

　　(一)积极推动京津冀协同发展。

　　坚持把推动京津冀协同发展作为全市工作的头等大事，认真落实《京津冀协同发展规划纲要》和《“十三五”时期京津冀国民经济和社会发展规划》，确保完成年度目标任务。

　　有序疏解非首都功能。严格落实2015版新增产业禁限目录，制定实施产业、市场、公共服务、行政事业单位四类非首都功能疏解方案，完成300家一般制造和污染企业退出任务，继续推进区域性专业市场的转移疏解和业态升级，推动部分市属高校和医院疏解。落实好非首都功能疏解示范项目，建立区域差别化公共服务及资源性产品价格形成机制，健全财政转移支付与落实功能定位、承接功能疏解、实施人口调控挂钩的机制，加快疏解步伐。

　　以三个率先突破带动协同发展。认真落实京津冀交通一体化规划，加快推进京张铁路、京唐城际、京台高速、延崇路等项目建设。继续实施京津风沙源治理、太行山绿化工程，新增10万亩京冀生态水源林，持续治理永定河、潮白河、拒马河等跨界河流。积极搭建“4+N”功能承接平台，加快推进曹妃甸首钢京唐二期等重大项目，抓好天津滨海-中关村科技园区建设，引导绿色产业项目向张承生态功能区布局。开工建设新机场外围交通和市政配套设施，支持机场周边村庄加快发展，推动临空经济区规划落地。

　　打造京津冀协同创新共同体。落实京津冀区域推进全面创新改革试验方案，开展知识产权、人才流动、激励机制、市场准入等方面协同性改革试验，争创具有示范带动作用的区域性改革创新平台。组建一批区域产业技术创新战略联盟，推动创新资源开放共享。围绕科技冬奥、生态安全、服务民生等领域实施协同创新工程，开展关键技术联合攻关和集成应用。用好中关村协同创新投资基金，加快张北云计算产业基地等园区建设，推动形成科技创新园区链。

　　扎实做好脱贫帮扶和对口支援工作。认真贯彻中央扶贫开发工作会议精神，加快制定对口帮扶河北贫困地区发展的落实方案，安排相关区开展对口帮扶，帮助贫困县改善生产生活条件。继续做好援疆、援藏、援青等工作，实施好产业、教育等领域援助项目，完成京蒙对口帮扶年度任务，做好南水北调水源区对口协作。研究探索环渤海地区合作发展协调机制，落实跨区域重大基础设施建设等六大方面重点任务。坚决贯彻中央全面振兴东北地区等老工业基地的若干意见，积极推动区域合作发展。

　　全力做好2022年冬奥会筹备工作。坚持绿色办奥、共享办奥、开放办奥、廉洁办奥，制定筹备工作总体规划，确定路线图和时间表。编制场馆和基础设施总体规划，确定新建场馆招标和设计方案，推动基础设施建设，启动冬奥会市场开发。同步做好冬残奥会筹备工作。制定加快冰雪运动发展的意见，科学规划北京冬季竞技体育项目布局，推动冰雪运动普及发展，营造全社会关心、支持和参与冬奥的良好氛围。

　　(二)全面深化改革开放。

　　坚持问题导向，扭住关键、精准发力、狠抓落实，以深化改革破解制约发展的瓶颈问题，以扩大开放拓展发展空间。

　　深化行政审批制度改革。深入推进简政放权、放管结合、优化服务，做好中央精简行政审批事项的衔接落实，完善权力清单、责任清单制度。加强事中事后监管，加快社会信用体系建设，建立登记注册、行政审批、行业管理相互衔接的协同监管机制。深化商事制度改革，推行工商登记全程电子化服务，探索开展市场主体简易注销登记试点。

　　深入推进经济领域重点改革。持续推进投融资体制改革，创新投资运营模式，建立和完善合理投资回报机制，引导社会资本扩大投资。深化预算管理制度改革，完善均衡性转移支付政策，建立大额专项资金统筹管理机制，加强政府债务限额管理。进一步理顺供热价格，分区域调整非居民水价、电价。完善国有企业改革制度体系，做好国有资本投资、运营公司改组等试点工作，推动一级企业调整重组和整体上市，积极稳妥发展混合所有制经济。全面落实促进中小企业发展条例，完善中小企业公共服务平台网络，支持非公有制经济发展。

　　更加积极主动扩大开放。强化国际交往功能，完善重大活动服务保障常态化机制。主动融入“一带一路”建设，积极推进国际产能和装备制造合作，完善与亚洲基础设施投资银行、丝路基金等平台的对接机制。深化服务业扩大开放综合试点，滚动推出新的试点措施，完善服务业促进体系，带动科技、信息、旅游等服务业加快发展，提升“北京服务”品牌，努力形成可复制、可推广的经验。办好第四届京交会，扩大通信、金融、文化等新兴服务贸易。加快建设外贸转型升级示范基地和出口产品质量安全示范区，支持“双自主”企业扩大出口。鼓励企业扩大对外投资，带动装备、技术、标准和服务走出去。深入推进大通关建设，支持天竺综合保税区、平谷国际陆港建设，打造全方位口岸经济体系。扩大与港澳地区的经贸往来与合作，做好对台和侨务工作。

　　(三)大力推进创新发展。

　　坚持把创新作为引领发展的第一动力，深入实施创新驱动发展战略，加快建设全国科技创新中心，构建“高精尖”经济结构，实现更高质量、更高效益的发展。

　　强化中关村示范区创新引领作用。围绕打造国家自主创新重要源头和原始创新主要策源地，深化科技体制机制改革，着力激发企业创新、科研机构和高校创新、市场转化科技成果“三方面潜力”。一是深入推进先行先试改革。推动出台新一批支持中关村全面创新改革的试点政策，加快落实科技成果使用、处置和收益管理改革。二是优化创新空间布局。完善“一区多园”统筹发展机制，探索园区新型管理运营模式，推进中关村科学城、未来科技城、怀柔科学城建设发展。聚焦中关村核心区，高水平建设中关村大街。三是强化企业创新主体地位和主导作用。制定鼓励企业研发投入政策，开展龙头企业创新改革试点，促进大企业与小企业创新对接。支持产业技术研究院、中关村开放实验室等平台建设，推动央地、军民、高校院所协同创新。四是提升原始创新能力。深入实施技术创新行动计划，加快实施12个市级重大科技专项，支持各类创新主体承接国家重大创新任务，突破一批核心、关键和共性技术。健全科技成果转化应用激励机制，推动创新成果资本化、产业化。

　　打造大众创业、万众创新新引擎。落实大众创业万众创新行动计划，支持众创、众包、众扶、众筹发展。鼓励发展投资促进、培训辅导、媒体延伸等创新型孵化器，打造特色鲜明的众创空间。落实加快建设国家科技金融创新中心的意见，构建科技金融一条龙服务体系。推动出台中关村互联网金融综合试点方案，开展股权众筹融资试点，加强互联网金融监管。深化中关村人才管理改革，完善人才评价机制，建立以能力、业绩和贡献为导向的人才评价体系，加快建设国际人才港。鼓励高等学校新设科技成果转化岗位，支持科技人员创业。加强知识产权保护，推进标准化建设，营造公平竞争的创新创业环境。

　　培育内需增长新动力。准确把握去产能、去库存、去杠杆、降成本、补短板五大任务，着力加强供给侧结构性改革，扩大有效供给，以新供给创造新需求。完善绿色消费政策，扩大节能减排产品、新能源汽车、有机农产品等绿色消费。鼓励信息消费终端升级换代，加快发展移动互联网、数字电视等信息服务。持续提升生活性服务业品质，推进便利店、早餐、蔬菜零售等8项基本便民服务在城六区社区全覆盖。增加多元化养老健康供给，壮大养老健康消费。实施旅游消费提升计划，做优古都文化、京郊休闲等旅游板块，扩大外来消费。保持有效投资力度，着力补齐基础设施短板，重点投向水环境、大气、交通、棚户区改造、养老等领域，抓好市重点工程，提高投资的精准性。进一步拓展资金筹集渠道，大力推广政府和社会资本合作模式，推动试点项目加快落地。

　　推动高端产业加快发展。支持金融业创新发展，加快发展现代保险服务业，促进“新三板”、“四板”健康发展，规范完善要素市场。扩大信息服务业优势，加快云计算平台建设，积极发展大数据产业。聚焦研发服务、知识产权服务、检验检测认证服务等领域，拓展科技服务业市场空间。实施《中国制造2025》北京行动纲要，用好高精尖产业发展基金，组织实施八个新兴产业专项，力争在新能源汽车、集成电路、机器人、3D打印等重点领域取得突破。开展绿色制造技术改造行动，推动传统产业加快升级。深入实施“互联网+”行动，围绕城市交通、公共安全、生态环境等15项重点领域，推进一批示范项目。贯彻落实军民深度融合发展战略，推动军民融合产业园建设发展。优化“高精尖”产业空间布局，研究制定工业用地二次开发利用一揽子政策，促进北京经济技术开发区等高端功能区内涵式发展。

　　(四)加快城乡区域协调发展步伐。

　　认真贯彻中央城市工作会议精神，扎实做好城市总体规划编制工作，落实“两线三区”全域空间管控机制，优化调整区域功能定位，构建平衡协调发展新格局。

　　推动老城功能重组。强化核心区政治活动、文化交流和国际交往等高端服务功能。制定更加严格的机动车使用、人口增长、企事业单位规模等管控措施，加快退出低端业态。落实核心区基础设施建设和环境改造提升方案，强化资金支持，推进老城区平房院落修缮改造和环境整治。优化老城用地结构，统筹利用疏解腾退土地，实现减人增绿，切实降低建设规模和开发强度，防止功能和人口过度聚集。

　　加快市行政副中心建设。突出绿色、宜居、人文、智慧发展，注重创业就业与居住功能均衡，加快形成内涵集约的发展模式，努力走出一条符合国情、有时代特色的副中心建设路子。完善市行政副中心规划方案，加快行政办公区起步区建设，确保到2017年市属行政事业单位部分迁入，带动其他行政事业单位及公共服务功能转移。扎实推进重点配套工程，完善交通联络路网，建成通州水厂，加快南水北调通州支线、配套电网、能源中心等设施建设。细化落实环球主题公园规划方案，全面推进园区主体工程开工建设。新建改扩建一批中小学、医疗机构和文化体育设施，增加优质公共服务资源供给。推进水环境治理，建设城市公园、湿地及公共绿地，改善生态环境，全面提升副中心城市品质。

　　切实增强新城承接能力。着力抓好顺义、大兴、昌平、房山等新城建设，推进城市更新改造和功能完善，积极承接中心城功能和人口疏解。其他新城要适度承接中心城功能疏解，重点吸引科研、教育、医疗机构入驻，带动所在区域城市化和城乡一体化发展。抓好国家新型城镇化综合试点，推动重点小城镇特色化发展。实施城乡结合部建设三年行动计划，深化“一绿”地区城市化建设试点，推动“二绿”地区城乡一体化建设试点，积极探索改造建设新模式。

　　深化农村改革发展。认真落实深化农村改革综合性实施方案，抓好集体资产股份权能改革试点，推进集体经营性建设用地入市试点工作，启动国有林场改革，全面开展土地承包经营权确权登记颁证。构建新型农业经营体系，发展多种形式的适度规模经营。加快农业调结构转方式，创建国家现代农业示范区。加强新型农村社区建设，完善乡村治理机制，完成第十届村委会选举工作。推进300个美丽乡村建设，启动农村基础设施“六网”改造提升工程，完成5.6万户农宅抗震节能改造等任务。继续加大对生态涵养区的扶持力度，打造生态服务型沟域经济带，搬迁山区险村险户7687人。认真做好第三次全国农业普查。积极筹办2020年世界休闲大会。

　　(五)着力构建绿色发展新格局。

　　坚持生态优先，完善生态文明制度体系，着力解决生态环境突出问题，推动绿色低碳循环发展。

　　持续提升空气质量。完善大气污染防治责任落实机制，修订空气重污染应急预案，重点治理农村散煤、高排放机动车和城乡结合部污染，做好联防联控，巩固深化治理成效。实施400个村煤改清洁能源，完成3000蒸吨左右燃煤锅炉清洁能源改造。淘汰20万辆高排放机动车，提高公交、环卫、邮政等行业新能源车使用比例。深化治理城乡结合部，两年全部清退南部四区“小散乱污”企业。组织开展“大气污染执法年”行动，建立健全大气环境质量监测网络，严厉打击环境违法行为。

　　突出抓好水资源管理。实行最严格的水资源管理制度，全面推进节水型城市建设。做好南水北调江水调度保障和运行管理，加快黄村水厂等设施建设，启动农村饮水安全巩固提升工程，增强供水安全保障能力。实施水污染防治工作方案，落实新一轮污水处理设施建设三年行动计划，加大污水直排治理力度，提高污泥处理能力，防治农业面源污染。加强水源地保护，综合治理凉水河、清河等流域水系，着力解决支流沟渠“脏乱臭”问题，建设27条生态清洁小流域。大力实施雨洪利用工程，加快建设“海绵城市”。

　　着力扩展生态环境容量。新建城市绿地400公顷，提高公园绿地500米服务半径覆盖率。新增造林16万亩，加强平原造林后期管护，完成山区森林健康经营60万亩。完善资源有偿使用和生态补偿制度，推进全国生态文明先行示范区建设。加强土壤污染防治与修复。统筹山水林田湖生态养护，开展湿地保护与恢复工程。加快2019年世界园艺博览会重点项目和配套设施建设，启动招展招商和宣传推介工作，带动花卉绿色产业发展。

　　促进资源节约循环高效使用。强化约束性指标管理，实施能源资源总量和强度双控。加快发展节能环保绿色产业，建设资源循环利用体系。抓好公共建筑能耗限额管理，支持发展超低能耗建筑。完善生活垃圾分类与再生资源回收体系，严格餐厨垃圾和建筑垃圾收运管理，统筹推进垃圾处理设施建设，生活垃圾资源化率达到56%。大力倡导勤俭节约、绿色低碳，推动形成绿色发展方式和生活方式。

　　(六)坚决推进城市治理取得新进展。

　　坚持依法治理、共同治理，创新城市管理方式，提高城市治理能力，不断提升服务保障水平。

　　严格控制人口规模。坚持疏管结合、综合施策，落实人口调控措施，以功能疏解带动人口疏解，坚决遏制人口过快增长。大力整治地下空间和群租房，加强直管公房管理。持续开展“拆违打非”，拆除违法建设1500万平方米。出台实施居住证制度，落实积分落户政策。完善人口综合监测体系，提高人口调控预警能力。

　　加快治理交通拥堵。落实缓解交通拥堵行动计划，全力抓好建设、管理、服务，中心城绿色出行比例提高到71%。持续加大交通设施建设力度，开工建设2条新线，推进15条在建线路建设。加快兴延高速等项目建设，建成广渠路二期，完善中心城次干路、支路网络，完成100项疏堵工程。切实加强交通管理，降低机动车使用强度。完善差别化停车收费，开展停车治理专项行动，集中整治交通堵点、乱点，严厉查处交通违法行为。创新交通服务，增设50公里公交专用道，优化步行和自行车出行环境，新增公共自行车1万辆，提升交通智能化服务水平。

　　提高城市管理精细化、法治化水平。落实中央深入推进城市执法体制改革、改进城市管理工作的指导意见，提高管理、执法和服务水平。坚持重心下移、职能下沉、资源下放，强化街道统筹职能，加快构建“大城管”城市综合管理格局。推动网格化体系规范化建设，实现城市管理、社会服务管理、社会治安三网融合，广泛应用信息技术改善服务管理，建设智慧城市。加强城市服务管理标准化建设，做好试点示范。强化市政设施运行管护，推进地下综合管廊建设，完善气象灾害防御体系，提高防灾减灾和应急能力。下大力气治理城市痼疾顽症，综合整治100条街巷胡同，提升重点区域、重点大街景观水平，保持市容市貌整洁有序。

　　推进城市共建共享。创新双拥共建和优抚安置机制，支持国防和军队现代化建设，密切军政军民团结。认真贯彻全国城市民族工作会议精神，推进民族团结进步事业。贯彻落实党的宗教政策，切实维护宗教界合法权益。健全社区议事协商制度，推广参与式协商自治模式，完善“三社”联动机制，建设100个“一刻钟社区服务圈”。出台社会组织管理制度改革意见，建立混合登记体制，完善社会组织培育和监管体系，基本实现市级枢纽型社会组织服务管理全覆盖。壮大社会工作者队伍，推动志愿服务制度化。

　　(七)繁荣发展社会主义先进文化。

　　坚持社会主义先进文化前进方向，充分发挥全国文化中心的引领带动作用，当好文化繁荣发展的排头兵。

　　建设先进文化引领高地。深入开展中国特色社会主义和中国梦宣传教育，推动社会主义核心价值观融入到各个领域。深化群众性精神文明创建活动，广泛开展学雷锋志愿服务活动和公共文明引导行动，推进全民阅读，提高市民素质和城市文明程度。实施基层公共文化设施和服务提升工程，启动公共文化服务示范区创建工作，开展“深入生活、扎根人民”主题实践活动，推出更多文学艺术精品力作，更好满足群众文化需求。

　　传承弘扬优秀传统文化。实施文物保护工程，推进文保区腾退疏解和有机更新，加强长城文化带、“三山五园”等重点区域整体保护。推动中轴线申遗，着力打造“一轴一线”文化魅力走廊，加快建设前门历史文化展示区，逐步整体恢复天坛风貌。挖掘古都文化，传承京味文化，支持民族戏曲艺术、传统工艺美术发展，抓好经典出版、优秀典籍整理等工程，在城市建设中融入地域文化元素，发挥地方志资源在公共文化服务中的重要作用。加强非物质文化遗产保护，开展非遗代表性传承人抢救性记录工作，鼓励老字号品牌创新发展。

　　推动文化创意产业蓬勃发展。出台深化市属国有文化企业改革、加强国有文化资产监管两个意见，巩固扩大国有文艺院团改革成果，推动传统媒体和新媒体融合发展。制定实施文化创意产业发展指导目录，加大文化创意产业示范园区扶持力度，抓好国家文化产业创新实验区等示范基地建设。大力发展新兴文化业态，创建国家文化金融合作试验区，促进文化创意和设计服务与相关产业融合发展。

　　(八)不断提升民生保障水平。

　　坚守底线、突出重点、完善制度，提供更公平更优质的公共服务，使全体市民在共建共享发展中有更多获得感。

　　努力促进城乡居民就业增收。做好新形势下就业创业工作，强化高校毕业生创业服务，带动青年就业创业。健全城乡劳动者终身职业培训制度，推行精细化就业服务和援助，加大就业困难群体、农村转移劳动力就业帮扶。实施失业保险支持企业稳定岗位政策，解决好结构调整中失业人员的再就业。全面落实中央关于打赢脱贫攻坚战的决定，调整低收入村、低收入农户认定标准，完善精准帮扶机制，努力促进低收入农户增收致富。

　　稳步提高社会保障水平。完善城乡居民养老保险制度，强化养老保险制度衔接，深化医保付费制度改革，稳步推进医保个人账户封闭管理，科学调整社会保障待遇标准。完善“救急难”工作机制，扩大医疗救助范围，实施困境儿童分类保障，健全扶残助残服务体系，提升无障碍设施建设管理水平。优先发展社区居家养老服务，落实新建小区和老旧小区配建养老服务设施政策，增加养老设施的有效供给，建设40个街乡镇养老照料中心，支持做好失能老人、重残老人护理，促进医养结合。抓好安置房源对接，完成3.5万户棚户区改造任务。建设筹集保障房5万套，加强保障房建设和使用管理，加大货币补贴政策实施力度，充分发挥市场配置资源作用，多种方式解决群众住房困难。

　　大力推动教育公平优质发展。全面推进素质教育，统筹配置义务教育资源，促进校长和教师在城乡、校际间合理流动，加强教师培训，加快数字学校建设，推动义务教育优质均衡发展。实施第二期学前教育三年行动计划，支持新建、改扩建幼儿园，多种方式增加学位供给。深化考试招生制度和教育教学改革，推动优质高中招生计划向普通初中倾斜。加快职业教育改革，开展高端技术技能人才贯通培养试验。提高少数民族教育发展水平。实施高水平人才交叉培养计划，推动高精尖创新中心建设，促进高等教育内涵式发展。

　　加快建设健康北京。落实食品药品安全三年行动计划，完善地方法规和标准体系，加大监管执法力度，强化食品药品安全供应保障，积极创建食品安全城市。深化公立医院综合改革，积极推进医药分开，实施药品阳光采购，合理调整医疗服务价格，完善分类补偿机制。深化全科医生执业方式改革，推广家庭医生式服务，发挥中医药在基层健康服务中的作用，加强农村医疗卫生服务机构建设，增强基层医疗机构服务能力。强化医联体内部分工协作，完善社区首诊、双向转诊的分级诊疗服务体系。健全传染病预防控制网络，优化急救网点布局，加强慢性病防控，提高公共卫生服务水平。落实“全面两孩”政策，优化妇幼保健和计划生育服务。实施“健康北京人”行动规划，积极开展全民健身活动。

　　确保首都和谐稳定。坚持把安全放在第一位，完善城市运行安全保障体系，确保城市运行安全有序。严格落实安全生产责任制，建立隐患排查治理体系，加强危化品和易燃、易爆物品安全管理，开展地铁、电梯、地下管线等重点领域专项整治，遏制重特大事故发生。落实重大决策社会稳定风险评估机制，强化诉访分离，促进信访与调解对接联动，抓好社会矛盾纠纷排查化解。加强网络空间管理，严厉打击非法集资。深化“平安北京”建设，完善立体化社会治安防控体系，加强反恐防恐能力建设，依法严厉打击各类违法犯罪活动，坚决维护和谐稳定的良好局面。

　　(九)进一步加强政府自身建设。

　　巩固拓展群众路线教育实践活动和“三严三实”专题教育成果，认真抓好“两学一做”学习教育，坚持依法行政、为民务实、廉洁高效，努力建设人民满意的服务型政府。

　　坚持坚定正确的政治方向。按照中央统一部署和要求，深入开展“学党章党规、学系列讲话，做合格党员”学习教育，切实增强政治意识、大局意识、核心意识、看齐意识、首善意识，坚持用中国特色社会主义理论的最新成果武装头脑、指导实践。严明党的政治纪律和政治规矩，坚决与以习近平同志为总书记的党中央保持高度一致，确保政令畅通。坚决贯彻中共北京市委的决策部署，以严的精神、实的作风，攻坚克难、干事创业，不断开创首都经济社会发展新局面。

　　全面推进依法行政。坚决执行市人大及其常委会的决议和决定，坚持重大事项报告制度，密切与政协委员、民主党派、工商联、无党派人士和人民团体的民主协商，认真办理议案建议和提案。落实国家法治政府建设实施纲要，出台我市实施方案，推动法治政府建设与创新政府、廉洁政府、服务型政府建设相结合。积极推进气象灾害防治等领域立法，制定排污许可证管理等政府规章，探索公众参与立法新途径。推动行政部门职责法定化，深化行政执法和监督体系建设，加强行政执法信息服务平台建设，抓好全国综合行政执法体制改革试点，严格规范公正文明执法，全面推行政府法律顾问制度。落实宪法宣誓制度，认真开展“七五”普法，健全媒体公益普法制度，增强全社会特别是公职人员尊法学法守法用法观念。

　　着力提高政府效能。健全重大行政决策程序，加强调查研究，支持新型智库建设，推进科学民主决策。创新公共服务提供方式，加大重点领域政府购买服务力度，引导社会力量参与。完善市政务服务中心功能，建设在线审批监管平台，探索实行“互联网+监管”模式。优化“督考合一”工作机制，严格绩效管理，严肃行政问责，提高政府服务质量与效率。推动行政行为公开，畅通社会投诉举报渠道，自觉接受社会监督。完善基层公务员考录政策，稳步推进公务员职务与职级并行，深入开展平时考核试点，提高公务员政治素质和业务能力。

　　坚持不懈改进作风。坚决贯彻落实十八届中央纪委六次全会精神，深入推进党风廉政建设和反腐败斗争。以基层和直接服务群众部门为重点，开展“不作为、乱作为”专项整治，坚决治理为官不为、懒政怠政。健全公共资金、国有资产、领导干部经济责任审计全覆盖制度机制，开展领导干部自然资源资产离任审计试点。继续保持惩治腐败高压态势，落实党风廉政建设主体责任，健全廉政风险防控机制，严肃查处以权谋私、失职渎职案件和腐败问题，切实做到干部清正、政府清廉。

　　各位代表！蓝图已绘就，扬帆正当时。让我们更加紧密地团结在以习近平同志为总书记的党中央周围，高举中国特色社会主义伟大旗帜，在中共北京市委坚强领导下，解放思想，开拓创新，攻坚克难，真抓实干，加快建设国际一流的和谐宜居之都，为实现中华民族伟大复兴中国梦而努力奋斗！
